# Crosstalk of Microorganisms and Immune Responses in Autoimmune Neuroinflammation: A Focus on Regulatory T Cells

**DOI:** 10.3389/fimmu.2021.747143

**Published:** 2021-10-07

**Authors:** Christina B. Schroeter, Niklas Huntemann, Stefanie Bock, Christopher Nelke, David Kremer, Klaus Pfeffer, Sven G. Meuth, Tobias Ruck

**Affiliations:** ^1^Department of Neurology, Medical Faculty, Heinrich Heine University Düsseldorf, Düsseldorf, Germany; ^2^Department of Neurology With Institute of Translational Neurology, University of Münster, Münster, Germany; ^3^Institute of Medical Microbiology and Hospital Hygiene, Heinrich-Heine-University Düsseldorf, Düsseldorf, Germany

**Keywords:** T cells, regulatory T cells, microorganism, pathogens, neuroinflammation, autoimmunity, microbiome, immunometabolomics

## Abstract

Regulatory T cells (Tregs) are the major determinant of peripheral immune tolerance. Many Treg subsets have been described, however thymus-derived and peripherally induced Tregs remain the most important subpopulations. In multiple sclerosis, a prototypical autoimmune disorder of the central nervous system, Treg dysfunction is a pathogenic hallmark. In contrast, induction of Treg proliferation and enhancement of their function are central immune evasion mechanisms of infectious pathogens. In accordance, Treg expansion is compartmentalized to tissues with high viral replication and prolonged in chronic infections. In friend retrovirus infection, Treg expansion is mainly based on excessive interleukin-2 production by infected effector T cells. Moreover, pathogens seem also to enhance Treg functions as shown in human immunodeficiency virus infection, where Tregs express higher levels of effector molecules such as cytotoxic T-lymphocyte-associated protein 4, CD39 and cAMP and show increased suppressive capacity. Thus, insights into the molecular mechanisms by which intracellular pathogens alter Treg functions might aid to find new therapeutic approaches to target central nervous system autoimmunity. In this review, we summarize the current knowledge of the role of pathogens for Treg function in the context of autoimmune neuroinflammation. We discuss the mechanistic implications for future therapies and provide an outlook for new research directions.

## 1 Introduction

In the context of infections, Tregs mediate beneficial and detrimental effects on short- and long-term disease outcomes. Although many Treg subsets have been described, thymus-derived (tTregs) and peripherally induced Tregs (pTregs) remain the most important subpopulations (1, 2). Tregs are generally found to express CD4 and either or both the high-affinity receptor for interleukin (IL)-2 CD25^+^ as well as the forkhead box protein P3 (Foxp3) (3). Their expression of intracellular and surface markers, such as CD25, glucocorticoid-induced tumor necrosis factor receptor (GITR) and the inhibitory cytotoxic T-lymphocyte-associated protein 4 (CTLA-4) define their phenotype and function (4–6). tTregs emerge with a CD4^+^CD25^+^Foxp3^+^ phenotype directly from the thymus. They are specific for self-antigens requiring continuous antigenic stimulation for their survival and preservation of self-tolerance, the lack of which may lead to autoimmune disorders (7–11). pTregs on the other hand adopt a regulatory function upon expression of Foxp3 and are therefore likely to be specific to an exogenous antigen (3, 12–16). In the context of infection, Tregs can ameliorate excessive immune responses by interaction with and suppression of immune cells. However, Treg expansion and enhanced Treg function are central mechanisms of pathogen-related immune evasion. Yet, the contribution of Tregs to the pathophysiology of pathogen-mediated diseases as well as the underlying molecular mechanisms remain largely elusive.

**Figure 1:**
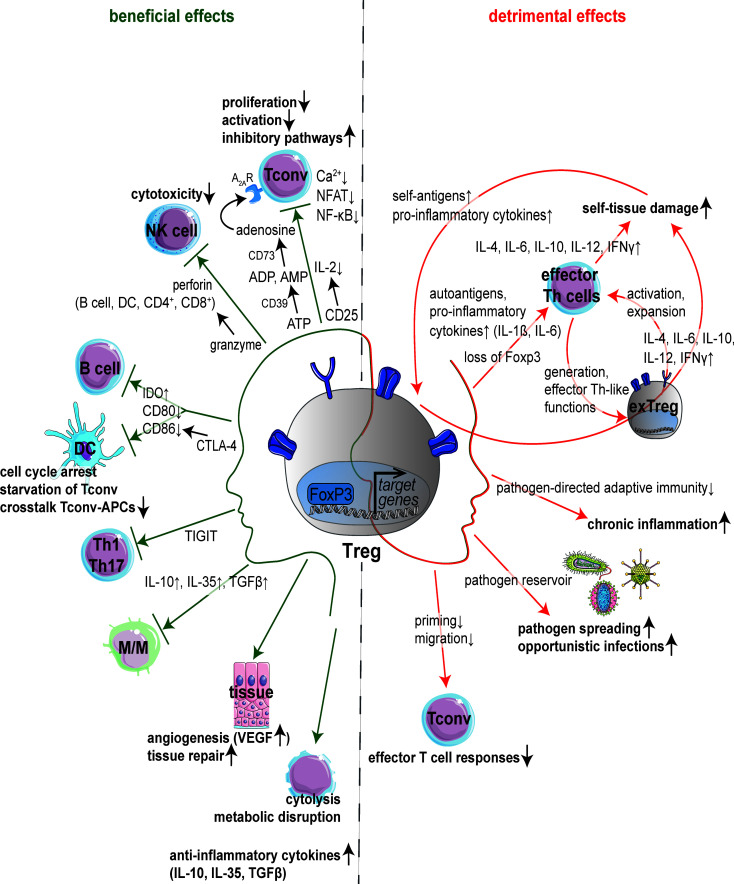
Janus-faced nature of Tregs. Schematic overview of molecular mechanisms underlying the regulation of immune cells and immune responses by regulatory T cells (Tregs). Anti-infective functions are mainly mediated by suppression of immune cells (left): Expression of CD25 leads to consumption of interleukin (IL)-2 inhibiting activation and proliferation of conventional T cells (Tconv). Suppression of Tconv can also be mediated by adenosine production *via* the ectoenzymes CD39 and CD73. Besides, Tregs are able to suppress T cell receptor (TCR)-induced Ca^2+^, NFAT and NF-κB signaling. Dendritic cells (DCs) and B cells are influenced by cytotoxic T-lymphocyte antigen 4 (CTLA-4) which binds CD80/CD86 and increases the expression of indoleamine 2,3-dioxygenase (IDO) resulting in starvation of Tconv next to cell cycle arrest and decrease in crosstalk between Tconv and antigen-presenting cells (APCs). Tregs can induce the death of effector cells (B cells, DCs, CD4^+^ and CD8^+^ cells) in a granzyme-perforin-dependent manner. The co-inhibitory molecule T cell immunoreceptor with Ig and immunoreceptor tyrosine-based inhibitory motif domains (TIGIT) suppresses T helper (Th) 1 and Th17 cell responses. Tregs can also induce angiogenesis *via* vascular endothelial growth factor (VEGF) or target tissue cells directly. Further immunosuppressive effects of Tregs are mediated by cytokines (IL-10, IL-35, TGF*β*), cytolysis or metabolic disruption. By contrast, Tregs can support inflammation (right) by a multi-layered feed-forward loop promoting the generation of ‘exTreg’ cells adapting Th-like functions, which in turn stimulate activation and expansion of autoreactive Th effector cells. Loss of immunosuppressive capacity adapting phenotype and functionality of Th cells is also reported upon loss of forkhead box protein 3 (Foxp3) in Tregs. Also, Tregs inhibit effector T cell responses thereby promoting chronic inflammation, pathogen spreading and opportunistic infections acting as pathogen reservoir. APCs, antigen-presenting cells; CTLA-4, cytotoxic T-lymphocyte antigen 4; DCs, dendritic cells; Foxp3, forkhead box protein 3; IDO, indoleamine 2,3-dioxygenase; IL, interleukin; M/M, monocytes and macrophages; TIGIT, T cell immunoreceptor with Ig and immunoreceptor tyrosine-based inhibitory motif domains; Tconv, conventional T cells; TCR, T cell receptor; TGFβ, tumor growth factor β; Tregs, regulatory T cells; VEGF, vascular endothelial growth factor.

In the context of therapeutic interventions, it is important to consider the Janus-faced functions of Tregs in infections potentially providing beneficial or detrimental effects **(****Figure 1****)**. Defining the mechanisms by which intracellular pathogens alter Treg function might pave the way toward new therapeutic approaches not only in the settings of infections, but also in autoimmune neuroinflammation.

**Figure 2:**
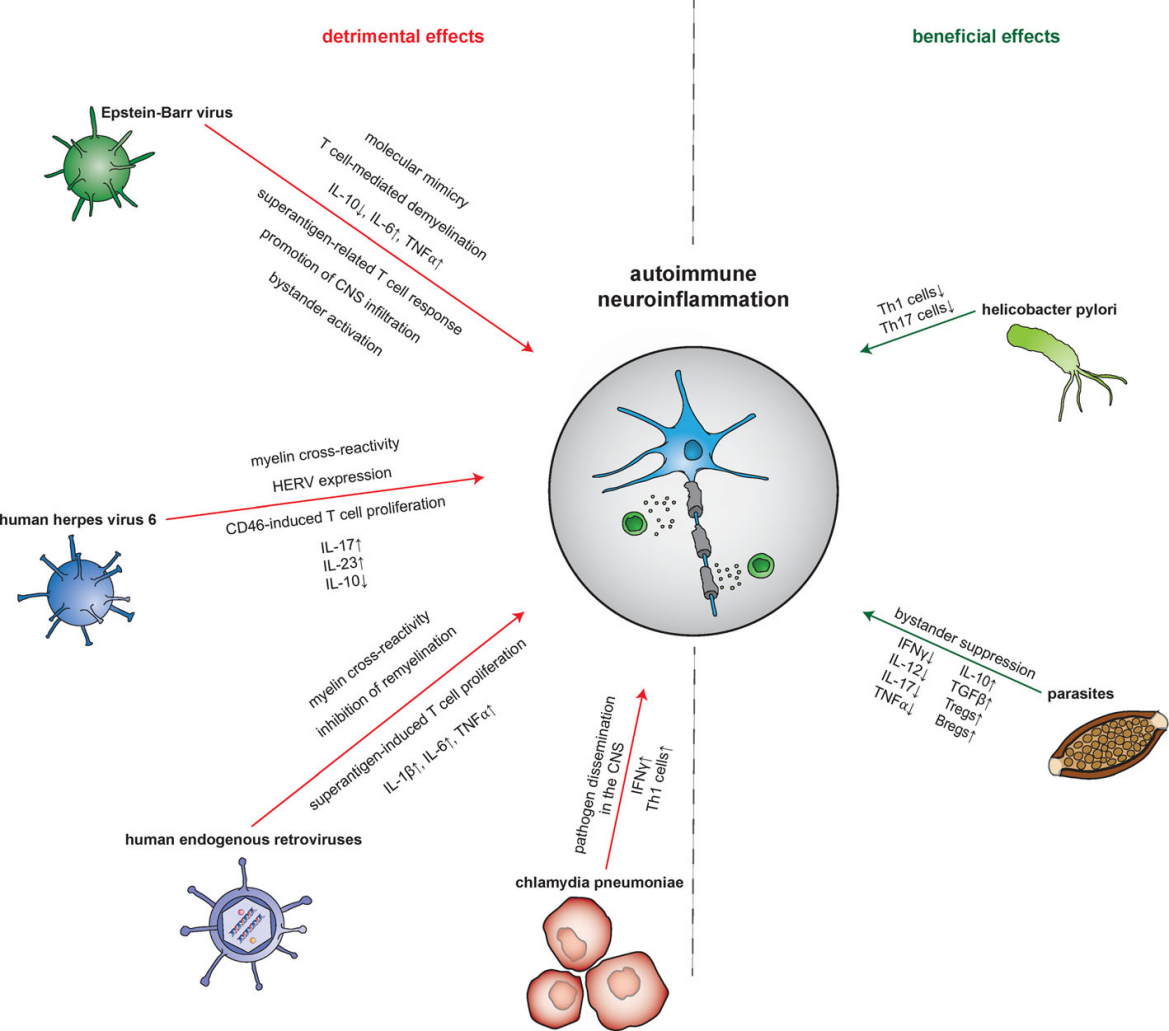
Pathogen-mediated impact on autoimmune neuroinflammation. The mechanisms by which infectious pathogens influence the processes of autoimmune neuroinflammation are diverse. Both detrimental and beneficial effects are reported. Epstein-Barr virus (EBV), for example, leads to an increase in neuronal damage *via* molecular mimicry, demyelination, an increase in pro- and decrease in anti-inflammatory molecules, and an augmented T cell response. Other pathways by which EBV induces amplification of the neuroinflammatory response include promotion of central nervous system (CNS) infiltration by autoreactive T and B cells next to bystander activation. Meanwhile, human herpes virus 6 (HHV-6) leads to a detrimental impact *via* CD8^+^ T cell-mediated cross-reactivity with myelin peptides and CD46-induced promotion of T cell proliferation. Furthermore, HHV-6 also triggers the expression of human endogenous retroviruses (HERVs) proteins. These in turn induce further damage *via* cross-reactivity with myelin antigens but also through acting as superantigens. Contributing to this is as well, HERVs trigger CD14- as well as Toll-like receptor (TLR) 4-mediated induction of proinflammatory cytokines. Interestingly, by suppression of oligodendrocyte precusor cells, HERVs also interfere with neurodegenerative processes. Finally, Chlamydia pneumoniae was shown to aggravate neuroinflammation in an animal model through pathogen dissemination into the CNS accompanied by an increase of pro-inflammatory Th1 cells. In contrast, a beneficial impact on the neuroinflammatory response was found for H. pylori and parasites. H. pylori improves the outcome in animal models of MS by reducing the proliferation of Th1 and Th17 cells. Parasites such as helminths attenuate the neuroinflammatory response by inducing bystander suppression *via* upregulation of regulatory B and T cells as well as anti-inflammatory cytokines. Bregs, regulatory B cells; CNS, central nervous system; HERV, human endogenous retrovirus; IFN, interferon; TGFβ, tumor growth factor β; Th cell, T helper cell; TLR, Toll-like receptor; TNFα, tumor necrosis factor α; Tregs, regulatory T cells.

**Figure 3:**
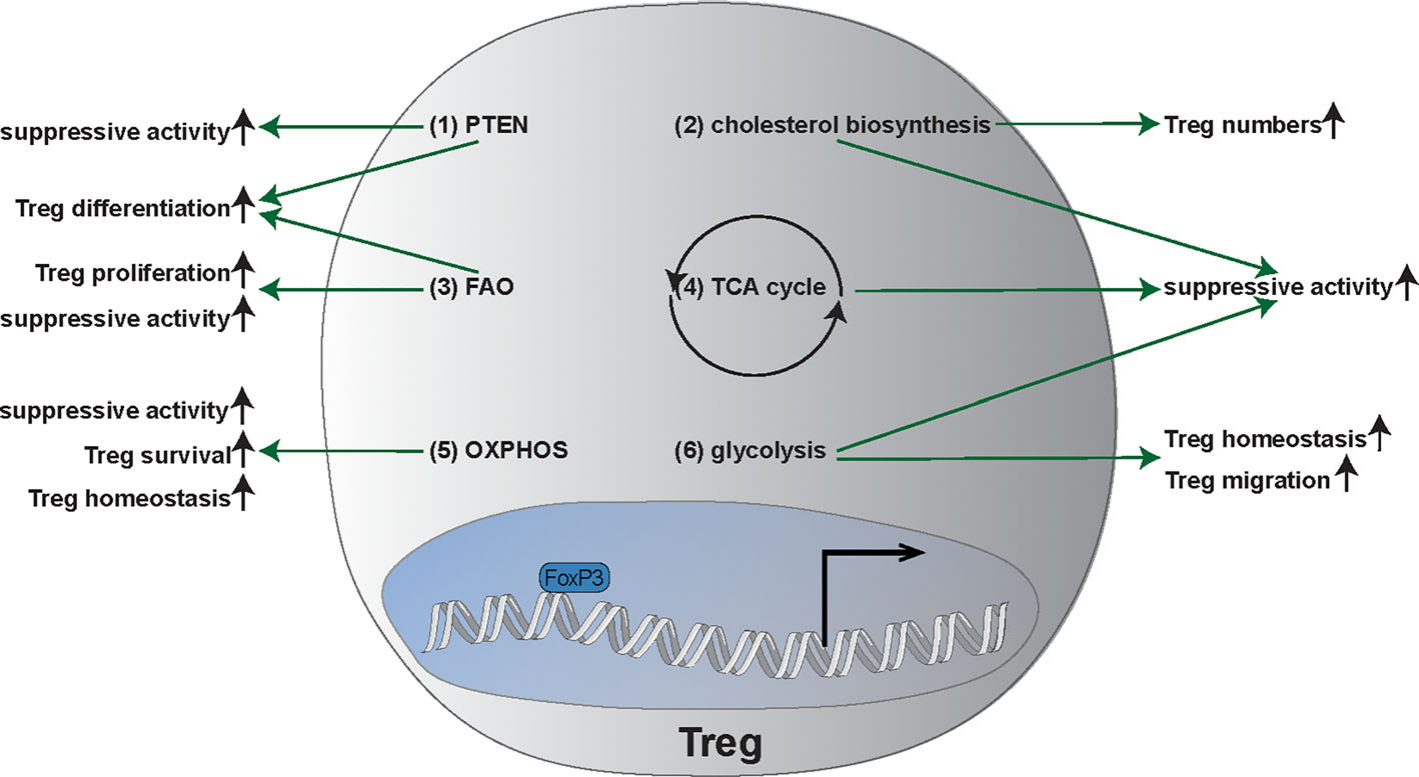
Main metabolic pathways and metabolic plasticity of Tregs. (1) Phosphatase and tensin homolog (PTEN) enhances regulatory T cell (Treg) differentiation as well as the suppressive activity of Tregs. (2) Cholesterol biosynthesis is required for suppressive functionality of Tregs and increases Treg frequency. (3) Fatty axid oxidation (FAO) is important for Treg generation, proliferation, as well as the suppressive activity. (4) Tricarboxylic acid cycle (TCA): TCA promotes the suppressive activity of Tregs. (5) Oxidative Phosphorylation (OXPHOS) is not only important for survival of Tregs but also for the suppressive capacity as well as the homeostasis of Tregs. (6) Glycolysis promotes suppressive function, the migration, and the homeostasis of Tregs. FAO, fatty acid oxidation; OXPHOS, oxidative phosphorylation; PTEN, Phosphatase and tensin homolog; TCA, Tricarboxylic acid cycle; Tregs, regulatory T cells.

With this review, we intend to give a detailed overview of molecular mechanisms underlying altered Treg function in models of acute and chronic infections as well as in autoimmune neuroinflammation with a focus on multiple sclerosis (MS) **(****Table 1****)**. We investigate the impact of pathogens on immune cell distributions, profiles, and functionality - particularly Treg functions - in the setting of neuroinflammation. We discuss how the complex changes in Tregs lead to altered function and that the underlying mechanisms could contribute to better understand the pathophysiology of neuroinflammatory diseases and their treatments. We further review the interplay of infection with pathogens and autoimmune processes **(****Figure 2****)** and, of particular interest, the clinical targets that result from these interactions (**Table 2**). In addition, we highlight the interplay between commensal bacteria and the function/plasticity of Tregs. In doing so, we particularly consider the implications for the phenotype of autoimmune phenomena. We point out the need for multi-omic approaches (functional analyses, transcriptomics, proteomics, and metabolomics) to illuminate the complex changes in Tregs leading to altered function (**Figure 3**).

**Table 1:** Impact of pathogens on Tregs and the underlying molecular mechanisms.

Infection	Pathogen *(human or murine)*	Molecular mechanisms	Impact on Tregs	Reference
**Viral**	Hepatitis C virus *(human)*	Production of TGFβ.	Treg induction.	(17)
	Herpes simplex virus 1 *(murine)*	Upregulation of HEVM, a binding site for viral glycoprotein HSVgD.	Treg expansion.	(18)
	Human immunodeficiency virus 1	Binding of gp120 to CD4 receptor of Tregs with consecutive upregulation of cAMP. Upregulation of CD39/adenosine axis and functional markers CTLA-4, TNFR, Foxp3, TGFβ, IDO. Increased expression of homing receptors CD62L and integrin alpha 4 beta in Tregs. Upregulation of Foxp3 expression in progressive infection.	Prolonged survival and higher suppressive activity. Accumulation in lymph nodes and mucosal lymphoid tissue.	(19–24)
	Friend retrovirus *(murine)*	IL-2 dependent: IL-2 production by FV-specific effector CD4+ T helper cells. Coregulation by B cells. IL-2 independent: Membrane bound TNFα binds to TNFR2. TNFR2 is indirectly upregulated upon FV infection.	Treg expansion.	(25–27)
	Human T-cell lymphotropic virus 1	HTLV-1 associated gene products inhibit Foxp3 expression.	Dysfunction of Tregs.	(28–30)
	Japanese encephalitis virus	Induction of PD-L1 on dendritic cells.	Treg expansion.	(31)
**Bacterial**	Mycobacterium tuberculosis *(human and mice)*	Induction of PD-L1 and CISH on dendritic cells.	Treg expansion.	(32, 33)
	Helicobacter pylori	Production of TGFβ. Upregulation of Foxp3 expression in children.	Treg induction.	(34, 35)
**Parasitic**	Plasmodium falciparum *(human and murine)*	TLR9 signaling. Burst of IL-2, IL-10 and TGFβ. Correlation between parasite expansion and Treg increase.	Treg induction and expansion. Upregulation of Foxp3 expression.	(36–39)
	Leishmania major	TGFβ enhances expression of integrin α_E_β_7_. Production of ligands for CCR5 by infected APCs.	Recruitment and retention of Tregs to infection site.	(40, 41)
	Toxoplasma gondii	Upregulation of GITR expression in Tregs.	Increased pathogen clearance and host resistance by enhancement of cellular immune responses.	(42)
**Fungal**	Candida albicans	TLR2 signaling.	Immunosuppression by increased IL-10 production and prolonged survival of Tregs.	(43)

APCs, antigen-presenting cells; CCR5, CC-chemokine receptor 5; CISH, cytokine inducible SH2-containing protein; CTLA-4, cytotoxic T-lymphocyte-associated protein 4; Foxp3, forkhead box protein P3; FV, friend virus; GITR, glucocorticoid-induced tumor necrosis factor receptor; HTLV-1, human T-cell lymphotropic virus 1; HVEM, herpes virus entry mediator; IDO, indoleamine 2,3-dioxygenase; IL, interleukin; PD-L1, programmed death-1 ligand 1; TGFβ, tumor growth factor β; TLR, Toll-like receptor; TNFR, tumor necrosis factor receptor; Tregs, regulatory T cell.

**Table 2:** Clinical studies on pathogen-derived targets in multiple sclerosis.

Target	Title	Therapy	Results	NCT	Reference
**Chlamydia pneumoniae**	Antibiotic Treatment Trial Directed Against Chlamydia Pneumoniae in Multiple Sclerosis	Rifampicin (300 mg twice daily) *vs.* azithromycin (500 mg every other day) *vs.* placebo	No effect on GEL. Reduction in brain parenchymal fraction loss.	NCT00043264	(44)
**EBV**	Hydroxyurea in Primary Progressive Multiple Sclerosis	Hydroxyurea (500 mg) *vs.* placebo	*Terminated (no efficacy in interims analysis)*.	NCT01103583	^*^
	Trial to Assess the Safety and Feasibility of Adoptive Cell Therapy with Autologous EBV-specific Cytotoxic T Lymphocytes (CTL) in Patients With a First Clinical Episode Highly Suggestive of Multiple Sclerosis (MS and EBV-CTL)	EBV-specific autologous cytotoxic T lymphocytes	*Still recruiting*.	NCT02912897	^*^
	Phase 1/2 Study to Evaluate the Safety and Efficacy of ATA188 in Subjects with Progressive Multiple Sclerosis (EMBOLD)	ATA188 (EBV-directed autologous cytotoxic T lymphocytes) vs. placebo	*Still recruiting*.	NCT03283826	^*^
	Tenofovir Alafenamide for Treatment of Symptoms and Neuroprotection in Relapsing Remitting Multiple Sclerosis	Tenofovir Alafenamide Fumarate (25 mg) *vs.* placebo	*Not yet recruiting*.	NCT04880577	^*^
	Phase I clinical trial of autologous Epstein–Barr virus-specific T cell therapy as treatment of progressive multiple sclerosis	EBV-specific autologous cytotoxic T lymphocytes (against EBNA-1 and LMP1, LMP2A)	Clinical improvement in 7/10 patients.	ACTRN12615000422527^#^	(45)
**HERV-W**	Safety Study of GNbAC1 in Multiple Sclerosis Patients	GNbAC1 (Temelimab; 2 mg/kg) *vs.* GNbAC1 (Temelimab; 6 mg/kg) *vs.* placebo	No safety concerns. Decline of HERV-W transcripts. 9 of 10 patients with stable MRI brain lesions.	NCT01639300	(46)
	Clinical Trial Assessing the HERV-W Env Antagonist GNbAC1 for Efficacy in MS	GNbAC1 (Temelimab; 6 mg/kg) *vs.* GNbAC1 (Temelimab; 12 mg/kg) *vs.* GNbAC1 (Temelimab; 18 mg/kg) *vs.* placebo	No reduction of GEL-T1 lesions after 24 weeks. Reduced new T1-hypointense lesions with 18 mg/kg GNbAC1. Consistent trends of reduced brain atrophy and magnetization transfer ratio decrease after 48 and 96 weeks.	NCT03239860	(47)
	Assessing the HERV-W Env ANtagonist GNbAC1 for Evaluation in an Open Label Long-term Safety Study in Patients with Multiple Sclerosis (ANGEL-MS)	GNbAC1 (Temelimab; 6 mg/kg) *vs.* GNbAC1 (Temelimab; 12 mg/kg) *vs.* GNbAC1 (Temelimab; 18 mg/kg)		NCT02782858	(47)
	Clinical Trial Assessing Temelimab Following Rituximab Treatment in Patients with Relapsing Forms of Multiple Sclerosis (ProTEct-MS)	GNbAC1 (Temelimab; 18 mg/kg) *vs.* GNbAC1 (Temelimab; 36 mg/kg) *vs.* GNbAC1 (Temelimab; 54 mg/kg) *vs.* Placebo	*Still recruiting*.	NCT04480307	^*^
	Raltegravir (Isentress) Pilot Study in Relapsing Multiple Sclerosis (INSPIRE)	Raltegravir (400 mg twice daily)	No effect on lesion load.	NCT01767701	(48)
**Helminths**	Helminth-induced Immunomodulation Therapy (HINT) in Relapsing-remitting Multiple Sclerosis (HINT)	Trichuris suis ova (2500 ova)	Reduction of new GELs compared to baseline. Reduced serum levels of IL-10.	NCT00645749	(49)
	Worms for Immune Regulation of Multiple Sclerosis (WIRMS)	Necator americanus larvae (25 larvae) *vs.* placebo	No reduction in MRI lesions. Increased numbers of Tregs.	NCT01470521	(50)
	Trichuris Suis Ova Therapy for Relapsing Multiple Sclerosis - a Safety Study (TRIMS A)	Trichuris suis ova (2500 ova)	No safety concerns.	NCT01006941	(51)
	Trichuris Suis Ova (TSO) in Recurrent Remittent Multiple Sclerosis and Clinically Isolated Syndrome (TRIOMS)	Trichuris suis ova (2500 ova) *vs.* placebo	*Clinical examinations could not be performed due to low patient compliance*.	NCT01413243	(52)

*Studies without PubMed-listed publication, extracted from ClinicalTrials.gov. ^#^Only Australian New Zealand Clinical Trials Registry number available. EBV, Epstein-Barr virus; EBNA-1, EBV nuclear antigen 1; GEL, gadolinium enhancing lesion; HERV-W, human endogenous retrovirus-W; IL, interleukin; LMP1, latent membrane protein 1; LMP2A, latent membrane protein 2A; MRI, magnet resonance imaging; MS, multiple sclerosis; Tregs, regulatory T cells.

Overall, our review focuses on past, present and future insights into the role of Tregs in the pathophysiology of pathogen-mediated diseases and in autoimmune neuroinflammation. We discuss established and potential therapeutic strategies in MS resulting from this new molecular understanding.

## 2 Manuscript/Subsections

### 2.1 Janus-Faced Nature: Duality of Tregs in Pathogen-Mediated Diseases

In the context of pathogen-mediated diseases, Tregs are Janus-faced **(****Figure 1****)**: On the one hand, they are the major determinant of peripheral immune tolerance not only controlling immune responsiveness to intrinsic antigens and infective pathogens but also modulating immune capacity (9, 11–13). On the other hand, Tregs dampen favorable pathogen-directed adaptive immunity counter acting complete pathogen clearance and giving rise to chronic infections (14, 53, 54). Their phenotype and functions are, among others, dependent on their localization and the tissue type (55–62).

#### 2.1.1 One Janus Face: Beneficial Treg Effects in Infections

Aside from their immunosuppressive capacity forestalling pathology, Tregs have been shown to facilitate appropriate effector mechanisms. Furthermore, Tregs control immunopathology detrimental to the host body arising from excessive effector immune responses.

Tregs utilize diverse immunosuppressive mechanisms depending on their microenvironment: Expression of CD25, the α-chain of IL-2, leads to consumption of IL-2 inhibiting activation and proliferation of conventional T cells (Tconv) (63–65). Interestingly, Chinen et al. showed that IL-2 expression activates signal transducer and activator of transcription (STAT) 5 further boosting the suppressor function of Tregs (66). Suppression of Tconv next to macrophages can also be triggered by cAMP-mediated protein kinase A (PKA) activation. Here, expression of the ectonucleotidase CD39 by Tregs leads to hydrolyzation of ATP followed by further cleavage of AMP to adenosine by CD73 (67–69). Subsequently, activation of the adenosine receptor A2A causes intracellular accumulation of cAMP which in turn stimulates PKA and associated downstream signaling (70–73). Tregs can induce the death of natural killer cells (NK cells) and other effector cells, such as B cells, dendritic cells (DCs), CD4^+^ and CD8^+^ cells, by releasing granzyme resulting in perforin-dependent apoptosis of target cells (74–76). B cells and DCs are regulated by Tregs *via* CTLA-4 which binds CD80/CD86 and increases the expression of indoleamine 2,3-dioxygenase (IDO) (77–79). Consecutively, binding of CD28 to CD80/CD86 is limited hampering the crosstalk between Tconv and antigen-presenting cells (APCs). Also, accumulation of IDO can lead to starvation of Tconv and cell cycle arrest, amongst others *via* degradation of the essential amino acid tryptophan (80, 81). Besides that, T cells can suppress T cell receptor (TCR)-induced Ca^2+^, NFAT and NF-κB downstream signaling (77). The co-inhibitory molecule T cell immunoreceptor with Ig and immunoreceptor tyrosine-based inhibitory motif domains (TIGIT) suppresses pro-inflammatory T helper (Th) 1 and Th17 cells, but not Th2 cell responses (82, 83). Tregs can directly induce angiogenesis *via* vascular endothelial growth factor or target tissue cells (58). Further immunosuppressive effects of Tregs, e.g. on monocytes and macrophages, are mediated *via* anti-inflammatory cytokines such as IL-10, IL-35 or tumor growth factor β (TGFβ), cytolysis or metabolic disruption (62, 77, 84).

During acute and chronic (retro-) viral infections, Tregs have been shown to promote the in- and efflux of pro-inflammatory cells into lymph nodes (85, 86). Also, they suppress the proliferation and entry of infected cells (87, 88) and contribute to a memory formation *via* antigen persistence (89). In mice, Treg response is locally defined controlling magnitude and duration of virus-specific cytotoxic T cell responses (90, 91). For example, in human and murine cytomegalovirus, vaccinia virus and influenza virus, CD8^+^ T cell responses are controlled by Tregs by suppression of the immune response to immunodominant epitopes (92, 93). In human immunodeficiency virus (HIV) low Treg frequencies are strongly associated with increased immune activation, accelerated atherosclerosis and other morbidities linked to inflammation (94–103). A negative correlation between the relative amount of Tregs and inflammation has also been observed for hepatitis C virus (HCV) in humans and mice (104, 105). Here, Tregs suppress not only the production of interferon gamma (IFNγ) but also the expansion and activation-induced cell death of HCV-specific T cells resulting in reduced CD4^+^ T cell reactivity and mitigation of T cell-mediated liver disease (105–109).

In bacterial infections, Tregs show a predominant regulatory function controlling and limiting adaptive and innate immune responses as shown in different mouse studies (110): Immunosuppressive functions of Tregs have been already described for helicobacter hepaticus (111, 112), helicobacter pylori (H. pylori) (113–115), listeria monocytogenes (116), pneumocystis carinii (117, 118).

In toxoplasmosis as a prototypical parasitic infection, Treg depleted mice showed 50-60% mortality during acute infection (119).

#### 2.1.2 Other Janus Face: Detrimental Treg Effects in Infections

Immune responses to pathogens can be impaired by an overly strong suppressive effect of Tregs interfering with early protective immunity (84): Tregs can inhibit effector T cell responses thereby promoting chronic inflammation. In turn, lack of complete eradication of pathogens leads to a reservoir function of human and murine Tregs acting as carriers for respective pathogens promoting their expansion in the environment resulting in spread of infections (120–122). Accordingly, pathogen clearance during the disease course correlates with a decrease in the suppressive capacity of Tregs (123). *Vice versa*, states of chronic inflammation are often characterized in humans by resistance to immune regulation by Tregs (124–126).

In friend retrovirus (FV) (127–130) and herpes simplex virus (HSV) infection in mice (54, 131, 132), Tregs limit CD8^+^ effector T cell proliferation and functions resulting in viral persistence. Treg expansion is mainly based on excessive IL-2 production by murine, FV-infected effector T cells (90, 91, 133, 134). In HIV, human Tregs inhibit effector T cell responses thereby promoting viral chronicity and opportunistic infections acting as a viral reservoir (**Figure 1**) (100, 135, 136).

In human and murine mycobacterium tuberculosis (Mtb) infection, expansion of Mtb-specific Tregs interferes with priming and migration of T cells to the infected lung resulting in deficient clearance of Mtb (**Figure 1**) (137–140). Here, Treg numbers inversely correlate with local mycobacteria-specific immunity. Three human studies have shown an increase in T reg numbers in the blood and at sites of infection during active disease (140–142). In human hepatitis B virus (HBV), the expansion of antigen-specific Tregs suggests their contribution to the liver pathology (143–145). Here, the frequency of Tregs correlates with viral load.

In murine fungal infections such as Candida albicans, the absence of Toll-like receptors (TLRs) and Tregs lead to an increase in immunity to candida *via* secretion of anti-inflammatory cytokines and improved leukocyte recruitment to infection sites (146, 147).

In parasitic infections [e.g., schistosoma in mice (148, 149), leishmania in humans and mice (150–155), plasmodium, and helminths (156, 157)], Tregs are reported to favor parasite expansion and persistence by limiting effector responses, especially of Th1 and Th2 cells, in an IL-10-dependent manner and by suppression of antigen-specific T cell proliferation (36, 158). Nevertheless, while Treg frequency correlated with parasite pathogen load, it also accounted for reduced liver pathology and improved host survival rates. Also, in murine chronic infections of the protozoan parasite *Toxoplasma gondii*, a nonresolving Th1 myositis occurs where Treg ablation during chronic infection rescues macrophage homeostasis and skeletal muscle fiber regeneration (159).

#### 2.1.3 Treg/Th17 Ratio in Pathogen-Mediated Diseases

In general, the balance between Th17 and Tregs is crucial for the maintenance of immune homeostasis during pathogen-mediated infections (160–162). By presentation of antigens *via* major histocompatibility complex II molecules on APCs and certain cytokine environments, naïve Th cells are activated and polarized into either peripherally-induced Tregs or Th17 cells to maintain homeostasis. Among APCs, macrophages are known to promote Treg responses, while DCs mainly activate Th17 cell responses (163). In mice, Th17 differentiation is mainly dependent on the cytokines IL-6 and TGFβ which induce the transcription factor retinoic acid-related orphan receptor gamma t (RORγt) in a STAT3-dependent manner (164, 165). In humans, Th17 differentiation mainly depends on IL-23 and IL-1ß (166–168). Th17 cell differentiation is further stimulated by TGFβ, TNF, IL-6, and IL-21. Maintenance and expansion of Th17 cells is regulated by IL-23 (168). Differentiation towards the Treg subset is stimulated *via* induction of the transcription factor STAT5 by TGFβ and in the absence of IL-6 (169–172). IL-2 and IL-10 also play important roles in the differentiation of Tregs (173). Th17 cells show pro-inflammatory effects during disease progression, which can result in autoimmunity. Tregs on the other hand, serve as immunoregulatory cells and maintain self-tolerance. These opposing effects are also represented on cytokine level: While Th17 cells secrete mainly IL17, IL-21 and IL-22, Tregs produce IL-10, IL-35 and TGFβ (160). *Via* IL-17 Th17 cells attract other immune cells, such as macrophages and neutrophils resulting in a state of chronic inflammation. In contrast, Tregs regulate differentiation and activity of Th17 and other T cells (8, 174). The Treg/Th17 ratio can contribute to a wide range of immune responses ranging from predominantly regulatory to stimulatory function. Its balance is crucial for immune homeostasis.

In recently infected SARS-CoV-2 patients, the number of Th17 cells was significantly increased compared to healthy controls causing inflammatory responses due to the production of pro-inflammatory cytokines (175, 176). In contrast, Treg numbers were decreased and even further downregulated in the disease course (177–179). Interestingly, the Treg/Th17 cell ratio and expression levels of their related cytokines were significantly higher in deceased patients than during remission (175).

Likewise, in respiratory syncytial virus infection but also in pulmonary infections in general, Tregs and Th17 cells have opposing features determining clinical severity (136, 177, 180, 181): Tregs promote viral clearance by recruiting CD8^+^ cytotoxic T cells to the lungs and limiting inefficient or excessive T cell responses (Th2, CD4^+^ and CD8^+^) (182–186). In addition, they control the innate immune response by neutrophils and NK cells. In contrast, Th17 cells hamper viral clearance by limiting CD8^+^ T cell responses and enhance Th2 immune responses resulting in a more severe clinical picture (187, 188).

In peripheral blood analysis of HBV patients, the Treg/Th17 ratio was decreased with reduced Treg levels and increased Th17 cell numbers (189). The latter correlated with TGFβ and IL-21 levels. Interestingly, here, the Treg/Th17 ratio was the best marker for predicting the stage of HBV-associated liver cirrhosis (190). In HCV patients, Th17 cells were associated with early infection and repair processes leading to liver fibrosis. Here, TGFβ and IL-10 suppressed Th17 cells (191).

For Mtb, the balance between Tregs and Th17 cells regulates encapsulation and control of lung lesions (192). If Tregs become predominant over Th17, Mtb disseminates more easily and recruitment of neutrophils to the infection sites gets delayed (192).

In gastrointestinal infectious diseases, Th17 are predominant in the acute phase producing the cytokines IL-17A, IL-17F and IL-22. In contrast, Treg/Th17 ratio increases in the chronic phase and infection progress because of the suppressive function of Tregs (193).

#### 2.1.4 Impact of Pathogens on Tregs

Several pathogens impact the immune status exploiting the regulatory T cell compartment to enhance their replication and become persistent (95, 194–196). While tTregs are usually specific for self-antigens requiring continuous antigen stimulation for their survival (8–11), pTregs are converted in the periphery and therefore more likely to be influenced by and specific for a microorganism-derived antigen (13, 14). Induction of Treg proliferation and enhancement of Treg function might be central to immune evasion mechanisms of intracellular pathogens (123, 197). In accordance, Treg expansion is compartmentalized to tissues with high viral replication (here Treg frequency often correlates with viral load) and prolonged in chronic infections (19, 25, 90). However, the molecular mechanisms by which intracellular pathogens alter Treg functions as well as the origin of these Tregs remain incompletely understood (for detailed overview see **Table 1**).

Some pathogens directly contribute to Treg induction. In humans, for example, hepatocytes infected with HCV or gastric epithelial cells infected with H. pylori induce Tregs *via* TGFβ production (**Table 1**) (17, 34). Upon infection with plasmodium falciparum there is a burst of IL-2, IL-10 and TGFβ associated with Treg induction and expansion. Here, Tregs were, among others, induced by TLR9 signaling in mice and expressed high levels of Foxp3 suppressing inflammatory processes and immunity-driving mediators of effector T cells (36–39).

Likewise, viral pathogens such as HSV-1, FV and Japanese encephalitis virus enhance Treg expansion (**Table 1**). In murine HSV-1, the viral binding site herpes virus entry mediator is upregulated (18). For FV infection, there are two possible mechanisms underlying Treg expansion: IL-2-dependent stimulation versus IL-2-independent, tumor necrosis factor (TNF) receptor 2-dependent upregulation (**Table 1**) (25–27). In both, Japanese encephalitis virus and Mtb, Treg expansion is a result of programmed death-1 ligand 1 (PD-L1) induction (31–33).

Further, pathogens can enhance Treg functions as shown in HIV infection. Here, upon binding of HIV glycoprotein 120 to the CD4 receptor, Tregs express higher levels of effector molecules such as CTLA-4, CD39 and cAMP and show increased suppressive capacity next to prolonged survival rates (**Table 1**) (19–23). Of note, expression of Foxp3 by patients with a progressive HIV-1 infection seems to be upregulated by individual T cells due to antigen stimulation. Moreover, Foxp3 expression in CD4^+^ T cells was shown to be a marker of HIV infection and potentially even a prognostic marker of HIV progression (24). Also, in the context of COVID-19 disease, an alteration in the expression of Foxp3 could be detected. More precisely, in patients with a severe disease course, a downregulation of Foxp3 could be detected in T cells indicating an impaired Foxp3-mediated feedback on T cell activation as potential mechanisms underlying disease progression (198). Similarly, human T cell lymphotropic virus 1 associated gene products are reported to inhibit Foxp3 gene expression thereby causing Treg dysfunction (**Table 1**) (28–30).

Interestingly, women infected with Chlamydia trachomatis displayed increased expression levels of Foxp3 in vaginal swab samples following the clearance of infection due to antibiotic treatment (199). In Candida albicans, prolonged Treg survival rates were achieved by TLR2 signaling and IL-10 production (**Table 1**) (43). Altered Foxp3 expression could also be detected in the context of parasitic infections: During chronic infection with Leishmania of the Viannia subgenus, a decreased Foxp3 expression was detected (200). Recruitment of Tregs to infection sites of Leishmania major was improved by expression of integrin αEβ7 and CC-chemokine receptor 5 (40, 41). Excitingly, infection with helminth parasites mediated by parasite-secreted proteins could also induce *de novo* T cell Foxp3 expression (201). This, in turn, may be a way through which parasites can evade the host immune response. Regarding H. pylori infection, it is worth noting that increased expression of Foxp3 on Tregs was observed in Tregs isolated from infected children, possibly contributing to an inverse association between H. pylori infection and allergic disease through changes in Treg functionality (35).

### 2.2 Role of Tregs in Multiple Sclerosis

Immune homeostasis and self-tolerance are regulated by the development, stability and function of Tregs (202). Tregs control immune capacity thereby influencing bystander immune responses such as allergies or autoimmune diseases (11, 14). Tregs prevent the activation and infiltration of T cells into the central nervous system (CNS) and maintain the homeostasis of the immune system (203–205). By suppressing CD8^+^ T effector cell responses, they limit parenchymal damage during CNS inflammation (206).

Tregs can contribute to the pathogenesis of autoimmune diseases by a multi-layered feed-forward loop (**Figure 1**) (84): Autoantigens and pro-inflammatory cytokines (IL-1β, IL-6 etc.) activate effector Th cells which further aggravate self-tissue damage by the expression of IL-4, IL-6, IL-10, IL-12 and IFNγ (207). Antigens and cytokines from damaged tissue promote the generation of ‘exTreg’ cells adapting Th-like functions which, in turn, stimulate the activation and expansion of autoreactive Th effector cells. These effector Th cell-like functions of ‘exTreg’ cells also directly stimulate pathogenic immune responses in local tissues and promote the pathogenesis of autoimmune diseases by participating in a feed-forward loop (**Figure 1**). Strikingly, dysfunctional, instable or insufficient Foxp3 expression can also trigger autoimmunity (15, 208–212): Upon loss of Foxp3, Tregs lose their immunosuppressive capacity adapting phenotype and functionality of Th cells (Th1, Th2, Th17), such as production of IFNγ and IL-17 (**Figure 1**) (213–215).

In contrast, CD4^+^ HLA-G^+^ tTregs cells were shown to ameliorate polyclonal adaptive immune response suppressing graft-versus-host disease *in vivo* (216). Likewise, Foxp3^+^ Tregs limit muscle destruction by cytotoxic T cells in dermatomyositis, polymyositis and inclusion body myositis (217).

Treg/Th17 imbalance is associated with autoimmune diseases such as MS, myasthenia gravis, psoriasis, inflammatory bowel diseases and rheumatoid arthritis (165, 218–223). Here, Th17 cells are regarded as the main driver of autoimmune inflammation activating other immune cells and secreting pro-inflammatory cytokines (224, 225). A decrease of Tregs in autoimmune and inflammatory diseases is reported to cause disease progression (226). Therapeutic approaches targeting the described Treg/Th17 axis are promising (227) and mainly aim at neutralizing Th17-secreted cytokines, reducing Th17 cell counts, increasing Treg cell levels and regulating transcription factors such as RORγt, STAT3 and Foxp3 (228–231).

#### 2.2.1 Qualitative Treg Alterations in MS

In MS pathogenesis, T cells acquire an autoreactive phenotype against CNS autoantigens followed by migration into the CNS causing inflammatory lesions. Activation of T cells is induced by molecular mimicry in the periphery or by autoreactive T cells in the CNS (232). Control mechanisms that should prevent autoimmunity, such as selection processes during tTreg development or peripheral suppression by Tregs, are often circumvented by autoreactive T cells (233, 234).

Tregs acquire a phenotype and expression profile resembling Th1 cells, thereby contributing to disease progression (235): Lower expression levels of Foxp3, TGF, CTLA-4 and CD39 were accompanied by an increase in IFN secretion in relapsing-remitting MS (RRMS) patients (236–238). Myelin-reactive T cells secreted high levels of IL-17, IFNγ and granulocyte-macrophage colony-stimulating factor compared to healthy controls (239). Next to an upregulation of markers associated with Th1 identity, Tregs expressed higher levels of the migration markers CD103 and CD49d enhancing transmigration of ‘exTregs’ into the CNS (238). High IL-17 levels have also been detected in the cerebrospinal fluid (CSF) of MS patients during relapse as well as in chronic lesions (222, 240), suggesting that both, the upregulation of IL-17 and down-regulation of Treg-mediated immunity, contribute to MS pathogenesis (241, 242).

Likewise, in experimental autoimmune encephalomyelitis (EAE), a mouse model of MS, an altered phenotype and impaired suppressive capacity of Tregs have been associated with clinical deterioration (235, 243, 244). Transfer of Th1-like ‘exTregs’ even lead to induction of EAE in naïve recipient mice. Interestingly, Othy et al. (245) showed that Tregs can suppress Th17 cells by inhibition of intracellular Ca^2+^ signaling and their contact to APCs.

Therefore, therapeutic induction of Tregs as well as modulation of Treg/Th17-related pathways could attenuate the inflammatory immune response resulting in mitigation of disease symptoms (246–250). Interestingly, Haas et al. showed that the immunosuppressive effect of Tregs after alemtuzumab treatment of MS patients was mainly due to an altered composition and reactivity of conventional CD4^+^ cells after immunodepletion (251).

#### 2.2.2 Quantitative Treg Alterations in MS

Treg frequency and the Treg/Th17 ratio were negatively correlated with disease severity in MS patients (252, 253). In RRMS patients, reduced Treg numbers were observed. In EAE, Treg plasticity was studied in detail showing an increase of ‘exTreg’ counts during the preclinical phase until disease maximum (212, 254). In line, remission is linked to an increase in Treg numbers representing a recovery of Treg identity (254). Interestingly, clinically isolated syndrome often preceding the diagnosis of MS was associated with alterations of the Treg compartment in peripheral blood (255) Here, patients displayed lower levels of immunosuppressive CD45RA^+^ Foxp3^low^ Tregs while levels of non-immunosuppressive CD45RA^-^ Foxp3^low^ Tregs and Th17-like Tregs increased. These observations suggest that progression to MS might be preceded by changes to the Treg compartment. A recent study investigating the mechanisms driving Treg dysfunction reported an inhibitory effect of circulating exosomes from MS on Tregs (256). This effect is thought to be mediated by let-7i miRNA interacting with insulin like growth factor 1 receptor and TGFβ receptor 1 expressed by CD4^+^ T cells. Thus, miRNA profiles from MS patients might directly inhibit Treg expansion (256).

### 2.3 The Interplay of Infections and Autoimmunity - Translation in the Setting of Multiple Sclerosis

It is commonly accepted that the interaction of genetic susceptibility and the exposure to certain environmental factors is crucial for the occurrence of autoimmunity. A major environmental factor contributing to the pathogenesis of autoimmune diseases and, more specifically, autoimmune neuroinflammation is pathogen-mediated infection. One of the underlying mechanisms is so-called molecular mimicry. Here, due to structural similarity of pathogen-derived peptides with host molecules, autoreactive B and T cells become cross-activated leading to an immune response directed against self-antigens (257). Likewise, epitope spreading is involved in the interplay of autoimmunity and infections. In this context, a new infection in an ongoing autoimmune disease leads to tissue damage with exposure of further self-antigens (258). APC-mediated presentation of these antigens to autoreactive lymphocytes then accelerates inflammatory processes (258). Furthermore, infections can facilitate inflammatory processes through bystander activation leading to a general immune response with activation of immune cells such as NK cells or macrophages and thus release of pro-inflammatory cytokines (259). This inflammatory milieu induces an antigen-independent activation of primed B and T lymphocytes at the inflammation site and thereby enhances autoimmune damage. Lastly, pathogen-mediated amplification of autoimmune events involves bacterial or viral superantigens leading to an extremely potent activation of polyclonal autoreactive T cells by binding to major histocompatibility complex II (258). These superantigens lead to a massive proliferation of T lymphocytes with excessive cytokine production, especially of IL-2 and IFNγ, resulting in an exacerbation of autoimmune processes (258).

The Treg compartment is needed to control immunopathology throughout life. However, while Tregs are indispensable for immune regulation, exuberant Treg function might prove detrimental for host defense. For example, in Mtb as a model for chronic bacterial infection, Tregs can delay leukocyte migration from lymph nodes to sites of ongoing infection (107, 260). In line, Treg ablation reduces accumulation of Mtb in lungs of infected mice (139). These observations underpin the potential of Tregs to exert detrimental effects in immune-mediated diseases. The continuum of dysfunctional Treg action is shared between infectious conditions, characterized by exuberant immunosuppression, and autoimmune conditions, characterized by promotion of immunogenicity (84). Common to Treg dysfunction is the instability of Foxp3 (213). Foxp3 is pivotal for Treg homeostasis. However, lineage tracing studies revealed that Foxp3 is frequently lost under autoimmune conditions (213). Loss of Foxp3 leads to generation of the so called ‘exTreg’ phenotype characterized by functions shared with effector Th cells, such as secretion of pro-inflammatory cytokines e.g. IFN-γ and IL-17 (84). Moreover, continuous IL-2 signaling is needed to prevent loss of Foxp3 (261). Intriguingly, inflammatory conditions promote loss of Foxp3 and, therefore, contribute to maintaining autoimmune states (212). Inflamed tissue constitutes a complex micro-environment characterized by immune cell infiltration, pro-inflammatory cytokine secretion and increased (self-)antigen presentation. Treg instability might therefore contribute to sustaining inflammatory conditions whereas inflammation promotes loss of Foxp3 and generation of Tregs more closely resembling effector Th cells further contributing to pro-inflammatory stimuli in a feed-forward loop (84). Intriguingly, Tregs from patients who resolved an HCV infection reacted to a virus-encoded peptide with substantial human homology while Tregs from non-infected patients did not (260). Taken together, the pathogenic potential and lineage instability of Tregs make them suspects for mediating autoimmunity following chronic infections.

One of the best studied pathogens involved in MS pathophysiology is Epstein-Barr virus (EBV). Based on epidemiological similarities, an association of EBV infection and MS was suspected early on (262). Further research subsequently not only proved that virtually all MS patients exhibit an EBV infection (263), but also that prior infectious mononucleosis is associated with a 2-3 times higher risk of developing MS (264). Conversely, this risk is significantly reduced for individuals with a negative EBV serology (265). Particularly interesting in this context are data showing that initially seronegative patients experience seroconversion shortly before the onset of MS symptoms (266). Even in pre-symptomatic patients with EBV, a significant increase in anti-EBV antibodies was found over five years before disease onset suggesting involvement of EBV in early disease stages (267). Interestingly, since an association between EBV serology and early conversion of clinically isolated syndrome into clinically definite MS has been demonstrated (268), EBV serology also appears to correlate with disease activity. Consistently, a correlation between anti-EBV nuclear antigen 1 (EBNA-1) titers, disease progression, lesion load, brain atrophy, and the extent of demyelination in MS patients has been demonstrated (269–271). Further support for an involvement of EBV in MS pathophysiology derives from histological studies revealing an accumulation of EBV-infected B- and plasma cells in MS brain meninges, in cortical as well as in white matter lesions (272–274).

Despite this overwhelming evidence, the molecular mechanisms underlying the role of EBV in the immunopathophysiology of MS are still not properly understood. However, a number of hypotheses exist involving discussion of both modulation of peripheral and CNS-localized immune responses. In the periphery, EBV might lead to a cross-activation of pathogenic T cells *via* molecular mimicry as described above (275). This theory is supported by the fact that EBNA-1-specific T cells react to myelin antigens more frequently than to other auto-antigens causing a release of pro-inflammatory IFNγ (276). Furthermore, EBV-reactive T cells were isolated from the CSF of MS patients also recognizing myelin basic protein (MBP) (277). In addition, a very recent study found cross-reactivity between EBNA-1 and the recently identified MS autoantigen called anoctamin 2 further supporting EBV-induced molecular mimicry (278). Another hypothesis proposes that EBV infection of peripheral B cells induces the expression of aB-crystallin. As it is also expressed in oligodendrocytes, an aB-crystallin-directed T cell response might ultimately lead to demyelination (279). Furthermore, there is evidence for EBV infection of B cells leading to a release of predominantly pro-inflammatory cytokines such as IL-6 or TNFα and simultaneously impeding immunoregulatory processes by reducing IL-10 levels (280, 281). Further evidence derives from recent data providing another pathophysiological link between EBV infections and MS. Wang et al. reported an autoreactive CD4^+^ T cell clone showing cross-reactivity between HLA-DR-derived self-proteins, EBV antigens, as well as autoantigens presented by HLA-DR allomorphs DR2a and DR2b (282). Thus, EBV antigens could be actively involved in the activity of autoreactive CD4^+^ T cells. Since HLA-DR15 is one of the genetic factors most strongly associated with MS, this link highlights the relevance of EBV infection in the pathogenesis of MS (282). A theory with regard to the modulation of peripheral immune processes describes that the invasion of autoreactive T- and EBV-infected B cells into the CNS is forced by expression induction of EBV-induced G protein-coupled receptor 2 thereby fostering the neuroinflammatory response **(****Figure 2****)** (275, 283–285).

In the CNS, the accumulation of infected B cells within the meninges and perivascular cuffs suggests that these B cells may elicit a CD8^+^ T cell response, leading to a multiplication of the inflammatory response *via* bystander activation (272, 286). Expression of superantigens by EBV-infected B cells could further lead to an excessive T cell response (287). Finally, it is hypothesized that EBV-induced immortalization of infected B cells and exhaustion-induced defective elimination lead to an accumulation of EBV-infected autoreactive B cells causing a permanent exposure to CNS antigens (**Figure 2**) (288–290). This exposure might considerably aggravate CNS damage in the context of autoimmune neuronal inflammation by antigen expression, autoantibody production, as well as by providing survival signals to autoreactive T cells.

Besides EBV, human endogenous retroviruses (HERVs) seem to be significantly involved in MS pathophysiology. These proviruses which account for circa 8% of the genome originate from exogenous infection of primate germ line cells millions of years ago and are today part of the human DNA (291). While they are functionally inactive under physiological conditions, pathological triggers such as viral infections can induce reactivation and thus production of viral proteins (292). First evidence of an involvement in MS dates back more than 30 years when retrovirus transcription was found in the supernatants of meningeal cell cultures of MS patients (293). Different HERVs such as HERV-H, HERV-K and HERV-W were subsequently associated with MS (294, 295). In addition to an increased HERV-W expression in MS patients (296), observations of higher antibody reactivity to certain HERV-W sequences in MS patients (297) and HERV-W upregulation in MS plaques correlating with disease activity support the involvement of HERVs in MS (298). There is also clinical evidence of a relationship of HERVs and MS since patients expressing high levels of HERV-W show a poorer prognosis in early disease stages and increased disease progression (299). Accordingly, HERV-W load also correlates with disease activity and the occurrence of relapses (300). Once more, the possible underlying mechanisms are diverse. For example, reactivation of HERV-W proteins leads to an activation of both innate and adaptive immune responses in MS (301). Thus, dysregulated expression of HERVs may contribute to CNS damage such as observed in a severe combined immunodeficiency mouse model (302). This dysregulated HERV-W activity is likely to involve binding of its envelope protein (ENV) to TLR4 and its co-receptor CD14 which triggers the release of pro-inflammatory cytokines such as TNFα, IL-1β or IL-6 fostering the autoimmune response (303–305). Furthermore, HERV-W-derived proteins such as ENV show cross-reactivity with myelin antigens amplifying the neuroinflammatory response (306). Aside from immune-mediated mechanisms, HERV-W ENV also interferes with remyelination *via* the inhibition of oligodendrocyte precursor cell (OPC) differentiation (307). Moreover, it also induces a pro-inflammatory activation of myeloid cells which, in turn, contributes to axonal damage and thus neurodegeneration even in long-standing MS cases (308). As there is a monoclonal antibody available that neutralizes HERV-W-induced detrimental effects, endogenous retroviruses constitute an attractive target for future MS therapies (**Figure 2**) (47, 309).

There is an exciting connection between EBV and HERVs. Exposure of EBV-derived glycoprotein 350 to B cells, monocytes, macrophages, as well as astrocytes leads to a significant increase in the expression of HERV-W and syncytin-1 and is thus also associated with unfavorable processes (310). Similar to EBV, human herpes virus 6 (HHV-6) infection can trigger the expression of HERV-W as well as a HERV-related superantigen (**Figure 2**) (311, 312). HHV-6 is a neurotropic virus that is divided into two subtypes, of which subtype A can be found in oligodendrocytes of MS white matter lesions (313). In addition to the expression of HHV-6 antigens in MS plaques (314), further evidence for an involvement of HHV-6 in MS pathogenesis derives from elevated anti-HHV-6 antibodies in the CSF of MS patients, especially in patients with an exacerbated disease indicating HHV-6 as a trigger for disease aggravation (315–317). Interestingly, in a non-human primate MS-like animal model, prior infection with HHV-6 resulted in a worse outcome further supporting a detrimental impact of HHV-6 on MS (318). One of the HHV-6-mediated mechanisms contributing to MS pathophysiology involves molecular mimicry since cross-reactivity between HHV-6 and MBP was shown to induce cytotoxic T cell-mediated oligodendrocyte death (319). This idea is further supported by a close sequence homology between MBP and the HHV-6-derived U24 protein (320). Furthermore, it is suggested that HHV-6 binding to the CD46 receptor leads to a T cell-mediated autoimmune reaction (321). Also, increased IL-23 release by DCs and IL-17 production by T cells with a concomitant decreased secretion of the immunoregulatory IL-10 provide potential mechanisms of how also HHV-6 might exacerbate neuroinflammatory processes (**Figure 2**) (321).

Apart from viral infections, there is also evidence for the involvement of bacterial pathogens in MS pathophysiology. A large meta-analysis, for instance, has shown that MS patients have a significantly higher incidence of Chlamydia pneumoniae (C. pneumoniae) DNA and intrathecally synthesized immunoglobulins in their CSF compared to patients with other neurological diseases (322). In EAE, systemic infection of mice with C. pneumoniae led to dissemination of the pathogen into the CNS accompanied by an aggravation of autoimmune neuroinflammation through reduced Th1 cell proliferation as well as IFNγ production (**Figure 2**) (323). Nevertheless, the available data is still unclear and controversially discussed.

Whereas the pathogens mentioned so far all have a negative impact on the processes in MS, this is different for H. pylori. In MS cohorts for example, a reduced prevalence of the pathogen compared to controls was demonstrated (324). Even more, MS patients being seropositive for H. pylori showed reduced disability scores (325). In EAE, infection with H. pylori resulted in reduced disease progression, milder proliferation of autoreactive cells, and lower infiltration of pro-inflammatory effector Th1 and Th17 cells into the CNS (**Figure 2**) (326). A protective role of H. pylori is also assumed in other autoimmune diseases such as asthma (327) or inflammatory bowel disease (328).

Likewise, strong evidence for a protective role in MS disease development has been demonstrated for some parasites. First indications derive from epidemiological investigations, which showed an inverse relationship between parasites like Trichuris trichiura and the occurrence of MS (329). In fact, the prevalence of MS seemed to decrease when the contamination level exceeded 10% (329). Of note, parasite-infected MS patients showed a significantly decreased number of relapses, a minor decline in disability scores, and reduced magnetic resonance imaging (MRI) disease activity compared to patients without helminthic infection (144). Parasitic infections exert an anti-inflammatory effects both on the parasite-specific response and the inflammatory response directed against other antigens in the sense of bystander suppression (330). In mice, helminth infection significantly attenuated both the incidence and clinical symptoms of EAE (331, 332). This amelioration was accompanied by a decrease in pro-inflammatory IFNγ, TNFα, IL-17, and IL-12 with a simultaneous increase in the release of immunoregulatory IL-10 and TGFβ (330–332). Also in humans, helminth infection was associated with induction of CD4^+^CD25^+^Foxp3^+^ T cells suppressing the inflammatory response (333). Beyond Tregs, regulatory B cells secreting IL-10 were detected in greater numbers in helminth-infected individuals suffering from MS (334). Further, the MBP-specific immune response was characterized by a decreased release of pro- next to an enhanced release of anti-inflammatory cytokines in patients with a parasitic infection (**Figure 2**) (333). Interestingly, the protective effects of helminths infection were shown to be reversed following an anthelmintic treatment concerning both the clinical as well as radiological MS activity and the immunosuppressive effects in terms of the Treg activity (335, 336).

In summary, for many pathogens there is versatile evidence for modulation of autoimmune processes in the context of neuroinflammation in MS. Nevertheless, it has not yet been possible to conclusively define the underlying molecular mechanisms.

#### 2.3.1 Therapeutic Targets

The above findings on detrimental but also beneficial effects of pathogenic infections have led to therapeutic approaches - in some cases despite continuing doubts about the mechanistic background.

In the case of EBV, attempts have been made to prevent an acute EBV infection by prophylactic vaccination thereby reducing the risk for development of MS (274). However, there is currently no appropriate vaccination available. In general, antibodies directed against certain EBV proteins expressed during latency to increase anti-EBV immunity would be a promising strategy. Once again, however, no study results are available to date (274). In contrast, cell-based immunotherapies appear to be a more promising approach. In particular, the application of autologous or allogenic T cells targeting EBV-infected B cells came into focus. A first successful application of this therapy was already demonstrated in a patient suffering from secondary progressive MS (337). Subsequently, a study on the effects of a EBV-specific autologous T cell therapy using *in vitro* expanded T lymphocytes interfering with EBNA-1 and latent membrane proteins 1 and 2A was initiated (**Table 2**) (45). Clinical improvement occurred in 7 out of 10 included MS patients. Of note, this was only a safety trial therefore lacking a placebo group. Further studies elucidating the impact of autologous or allogenic T cells attacking EBV-infected B cells are underway (**Table 2**).

HERV-targeted therapies, on the other hand, have reached a more advanced stage. As already pointed out, HERV and HERV-related proteins such as ENV exert an unfavorable effect on OPCs and myeloid cells and thus on remyelination and neurodegeneration (307–309). In view of the persistent lack of remyelinating therapies (338), it is particularly interesting that this inhibition can be reversed by the anti-HERV-W IgG4 monoclonal antibody GNbAC1 (temelimab) (309). Besides promotion of remyelination, GNbAC1 impedes the release of pro-inflammatory cytokines (339). Application of GNbAC1 led to favorable effects in numerous early-phase studies (46, 340–342). Given these pre-clinical results, two phase IIa and IIb trials were initiated (**Table 2**) (47). Therapy with 18 mg/kg resulted in a significant reduction of the number of T1-hypointense lesions after 48 hours. Moreover, there was a consistent trend of reduced brain atrophy and a magnetization transfer ratio decrease indicating a positive impact on remyelination. However, GNbAC1 failed to achieve the primary endpoint of the study, i.e. reduction of gadolinium-enhancing lesions (GELs), possibly because of underdosing. However, the beneficial MRI effects on neurodegeneration raised hope and led to the initiation of a new phase II study (**Table 2**) (47). Another HERV-related approach is based on the theory that antiretroviral therapies can also induce inhibition of HERVs in MS (343). In a baseline-versus-treatment phase IIb study, 20 patients with active RRMS were treated with the integrase inhibitor raltegravir for 3 months (**Table 2**) (48). However, the primary study endpoint reduction in lesion load or development of new lesions during the treatment period compared with baseline was not met.

Although to date the evidence regarding C. pneumoniae is quite sparse, therapy with rifampicin or azithromycin for 6 months were compared with placebo in newly diagnosed RRMS patients with evidence of C. pneumoniae infection in the CSF (**Table 2**) (44). The primary endpoint, reduction of GELs, was not reached. Only a decrease in brain atrophy was found under antibiotic therapy. However, given the very small number of subjects, these results should be interpreted with great caution.

Recently, there are also first therapeutic approaches that exploit the protective effects of helminth infection on MS. In a small phase I study, MRI activity in five treatment-naïve RRMS patients was compared between baseline and after probiotic treatment with 2500 Trichuris suis ova every two weeks for three months (**Table 2**) (49). Under treatment, there was a reduction of new GELs by 70% compared to baseline with a return to baseline after two months of follow-up. The reduction of lesions was also associated with increased serum levels of of IL-4 and IL-10 in 80% of the participants (336). The follow-up study including 16 RRMS patients also showed a trend of reduction of active MRI lesions compared to baseline (344). Furthermore, there was an increase of Tregs observed during this trial. A safety study evaluating the effect of orally administered 2500 Trichuris suis eggs in 10 MS patients could not observe Trichuris suis ova-induced effects on disease progression (**Table 2**) (51, 336). In line, in this study there were no significant alterations detected regarding cytokine expression and T cell-specific transcriptions factors (336). A 9-month double-blind, randomized, placebo-controlled study enrolling 71 RRMS patients investigated the effect of transcutaneous application of hookworm larvae on lesion burden (**Table 2**) (50). Of note, treatment with hookworm larvae increased the proportion of Tregs in the peripheral blood. Furthermore, the study showed a tendency of reduced new or enlarging lesions as well as an ameliorated MRI activity in the treatment group. However, these differences were not significant (50). Given these inconsistent results together with methodological limitations such as small sample sizes, further studies are required to sufficiently address the therapeutic potential of helminth infections in MS (336).

### 2.4 Microbiome - the Missing Link Between Biomolecular Treg Signatures and Clinical Phenotype?

In recent years, the important role of the gut microbiome has been recognized in autoimmune diseases and pathogen-induced immune responses (345, 346). The interplay of the gut microbiome and the immune system may explain its seemingly universal impact on a great variety of diseases including autoimmune diseases, cancer, vascular disease, and even psychiatric disorders (347–350).

Importantly, a variety of factors modulate the composition of the microbiome. Hence, the relationship between the host and the microbiome needs to be understood as a dynamic rather than static process (351). One of the most influential factors of the microbiome is the diet, which under unfavorable conditions induces dysregulation in the form of dysbiosis (351). This dysbiosis, in turn, contributes to an increased incidence of gut-distal autoimmune phenomena such as autoimmune arthritis (352) or type 1 diabetes (353) through alterations in Treg/Th17 balance. In general, the dynamic balance or dysbalance of Tregs and Th17 is suggested to be a main effector mechanism by which the gut microbiome influences systemic immunity (354). Furthermore, antibiotic therapy has an enormous impact on the microbiome and thus on the function of CD4^+^ T cells. For example, antibiotic therapy not only leads to an altered colonic but also tTreg TCR repertoire (355). Likewise, antibiotic-treated mice show a significant reduction of Tregs in the colonic lamina propria (356, 357). Similarly, also germ-free mice exhibited a reduced Treg population (356, 358). However, restoration of the microbiome caused a strong re-expansion of Tregs in these mice (358). Importantly, the effects of an altered microbiome and thus altered Treg/Th17 balance expanded from the local milieu into the whole body. Consistent with this, it has been shown that gut-resident T cells traffic between different organs thereby exerting a systemic effect on the immune system (359, 360). In addition, a reduction of CD4^+^ memory T cells expressing the gut-homing chemokine receptor CC-chemokine 9 was detected in secondary progressive MS patients (361). These gut tropic CC-chemokine 9^+^ memory T cells were shown to exhibit a regulatory phenotype potentially contributing to the conversion of RRMS to secondary progressive MS (361).

Importantly, with respect to microbiome-mediated effects on immunoregulatory mechanisms, tTregs and pTregs need to be distinguished. Unlike tTregs, pTregs exhibit a TCR repertoire directed against microbiota and dietary antigens (362–364). In addition to pTregs and tTregs, other Treg populations such as latency-associated peptide-expressing Tregs, type 1 Tregs, RORγt-expressing Tregs, or GATA binding protein 3^+^ Tregs have been shown to play an essential role in mediating intestinal and extra-intestinal immune homeostasis (365).

This knowledge on the interplay of microbiome, Tregs and autoimmunity provides the rationale to investigate possible microbiome-associated therapeutic strategies such as a modified diet or use of probiotics to change Treg plasticity and function in a favorable way to treat autoimmunity. Therefore, the identification of the involved pathogens and signaling pathways is essential. One of the underlying mechanisms involves intestinal epithelial cell- and microbiota-mediated modulation of DCs leading to an immunoregulatory phenotype through upregulation of IL-10, TGFβ and retinoic acid (365). The potent induction of colonic Tregs by Clostridium species has been well studied. Colonization of germ-free mice with a composition of 46 Clostridium strains resulted in a marked expansion of Tregs (356). Matrix metalloproteinases induced by Clostridium were involved in this expansion and promoted the differentiation and survival of Tregs *via* TGFβ. Furthermore, Clostridia are known to enhance levels of short-chain fatty acids (SCFAs) (366) which effectively stimulate Tregs. For example, in contrast to long-chain fatty acids that promote a Th1 as well as Th17 response, SCFA administration induces a reduction in the number of Th1 cells but an increased proliferation of gut Tregs (367, 368). Accordingly, application of SCFAs led to a significantly reduced disease progression in EAE and inflammation in ulcerative colitis (367, 369). Protective effects on type 1 diabetes in pre-diabetic mice as well as in a model of collagen-induced arthritis have also been described (368, 370). Interestingly, the SCFAs-induced effects appear to be mediated in part by epigenetic modifications *via* a suppression of histone deacetylases (371, 372). Despite these protective effects in several models of autoimmune diseases, SCFA application led to disease aggravation in an inflammatory model of rheumatoid arthritis (368). Therefore, a better understanding of the role of SCFAs and other microbiota metabolites is essential in order to exploit these findings therapeutically in clinical practice. In line with a significant impact of dysbiosis in the pathophysiology of MS, a Japanese study observed moderate dysbiosis in the microbiome of RRMS patients compared to healthy controls (373). Primarily, this involved a reduction of clostridial species as well as Bacteroidetes. Interestingly, however, the reduction did not affect the clostridial species, which is commonly known to have a protective effect on autoimmune processes by inducing colonic Tregs. This, in turn, underlines that deeper insights, especially into the influence of distinct species, are essential for translational approaches aiming to modulate the microbiome.

Additionally, several studies have demonstrated the relevance of Bacteroides fragilis, a human symbiont, for the function of Tregs (374, 375). By binding to TLR2, Bacteroides fragilis-derived capsular polysaccharide A as well as outer-membrane vesicles cause an immunosuppressive phenotype in terms of a significant expansion of IL-10-releasing intestinal Tregs (376, 377).

There is also evidence for a role of Akkermansia calcoaceticus and Akkermansia muciniphila in the context of autoimmune neuroinflammation. These bacteria induce impaired Treg conversion in peripheral blood mononuclear cells with concomitant stimulation of Th1 cell differentiation (378). Interestingly, in MS patients, a considerably increased occurrence of Acinetobacter and Akkermansia could be found, which, however, was decreased under MS therapy (351, 378, 379).

Furthermore, there is evidence of a DC-mediated Treg-promoting effect of Lactobacillus, Streptococcus, and Bifidobacterium (380, 381). Regarding potential therapeutic opportunities, application of probiotic mixtures consisting of these bacteria resulted in an expansion of Tregs (382). Therapy with Lactobacilli strains was also able to prevent disease progression in EAE by suppression of autoreactive T cells (383). In addition to these significant pre-clinical results derived from EAE, there is also human data underlining the impact as well as therapeutic potential of probiotic therapeutic approaches: In a small study including 9 MS patients and 13 healthy controls, probiotic therapy with Lactobacillus, Bifidobacterium and Streptococcus over two months led to a modulation of the microbiome as well as of the immune response (384). Thus, this therapy led to an enhancement of immunoregulatory processes with a reduction in the number of inflammatory monocytes as well as the expression of the costimulatory marker CD80 on monocytes. At the same time, probiotic therapy led to a decreased expression of the MS risk alleles HLA.DQA1 and HLA.DPB1 in control patients. However, no significant effects on peripheral Tregs were observed due to the intake of the probiotic treatment. On the microbiome level, several taxa known to be depleted in MS were increased whereas taxa associated with dysbiosis in MS were reduced (384).

Other interesting players influencing the activity and phenotype of bowel tropic Tregs are enteric neurons. Very recent data, for example, show that microbe-responsive Tregs in the colon lamina propria are localized in close proximity to nitrergic and peptidergic nerve fibers (385). Enteric neurons, in turn, prevent differentiation as well as the activity of Tregs *in vitro* by modulating IL-6. These processes, in turn, are decidedly influenced by microbial signals (385). Recent data describing a vago-vagal liver-brain-gut neuronal arc that induces proliferation and maintenance of pTregs further highlight the relevance of enteric neurons in the context of gut-tropic Tregs (386). Underlying mechanisms involve hepatic vagal sensory afferents that sense the gut microenvironment and on the other hand give input to vagal parasympathetic nerves as well as enteric neurons. Perturbation of the aforementioned afferent neurons led to a reduction in the frequency of pTregs. *In vivo*, this subsequently led to an increased symptomatology in animal models of colitis (386).

All these considerations have led to therapeutic approaches, such as antigen-based oral immunotherapies, which aim to achieve amelioration of autoimmune diseases *via* an expansion of Tregs induced by oral antigen administration (365). However, further studies on the critically responsible bacteria of the microbiome as well as the signaling pathways involved are required for a more widespread translational application.

### 2.5 Tregs in the Advent of the Era of Immunometabolomics

The large variety of pathogens, different transmission routes and complex underlying molecular mechanisms necessitate new comprehensive diagnostic approaches such as immunometabolomics. Several clinical studies addressing autoimmune inflammatory diseases like systemic lupus erythematosus, rheumatoid arthritis, Crohn’s disease and colitis ulcerosa, suggested that disease activity and outcome correlate with immunometabolic signatures (e.g. metabolite levels) (387–389). Moreover, Treg levels were found to be inversely correlated with body weight and body mass index (390). Altered Treg function is not restricted to adipose tissue but can be retrieved in flow cytometry analysis from circulating blood cells pointing to systemic effects (390). Thus, tissue-derived metabolic signals can exert systemic effects *via* local modulation of function and expansion of Tregs (391). This is particularly true for the adipose tissue. In the visceral adipose tissue of healthy mice, there is a significant enrichment of Tregs, whose deficiency leads to systemic consequences such as metabolic inflammation (60, 60, 392). In obese mice, for example, there is a reduction in the number of Tregs which, in turn, is associated with a decreased lipid content (60). Interestingly, at least in mice, there seems to be an inverse relationship between external lipid accumulation and intrinsic Treg lipid contents. In obese humans, on the other hand, a slight increase in visceral adipose tissue Tregs could be detected (393). Therefore, findings from murine experiments should not be carelessly extrapolated to humans, since underlying mechanisms may vary between species.

Next to the adipose tissue, liver-resident Tregs could also exert systemic effects on metabolic inflammation and systemic metabolism, depending on local metabolism and leading to the development of steatohepatitis or exacerbation of atherosclerosis upon deprivation (394, 395). Furthermore, hyperglycemia in diabetes poses a substantial risk for systemic infections disrupting lymphoid tissue integrity and affecting leukocyte development, phenotype and activity (396). Obesity and type 2 diabetes mellitus drive immune dysfunction, deteriorate infections, and increase sepsis mortality (397). Beyond the direct permissive effects of hyperglycemia on bacterial survival, our knowledge about molecular mechanism of immune deviation in obesity and diabetes remains fragmentary. T cells support coordinated immune responses with their metabolic flexibility **(****Figure 3****)** (398–401). Development, activation, function and maintenance of T cells are linked to their metabolic household and, depending on the phase, are associated with an altered metabolic state (399, 402–410).

Basal metabolism of Tregs is characterized by fatty acid oxidation (FAO), metabolism of acetyl CoA, and subsequently tricarboxylic acid cycle activity and oxidative phosphorylation (OXPHOS) (391). In contrast, during the induction of Tregs, there is a switch to a more glycolytic metabolism. This switch is characterized by a displacement of the glycolytic enzyme enolase-1 from the Foxp3 locus allowing the transcription of Foxp3 (411). Inhibition of glycolysis, e.g. using 2-deoxy-D-glucose, results in impeded Treg development due to lack of displacement of enolase-1 and ongoing Foxp3 inhibition (411). In contrast, multiple metabolic pathways are relevant for both homeostasis and proliferation of Tregs. Thus, Tregs show a constitutive activity of mechanistic target of rapamycin (mTOR), which, just like glucose transporter 1, showed an increased expression in proliferating Tregs (412). Interestingly, Procaccini et al. demonstrated that freshly extracted human Tregs show a very high level of glycolysis (413). Furthermore, Li et al. could show that both naturally occurring and tumor-derived Tregs cells exhibit significantly enhanced glucose metabolism as well as increased expression of glycolysis-associated genes further highlighting the role of glycolysis in this state (414). Besides glycolysis, oxidative metabolism has been shown to be critically involved in Treg homeostasis and expansion. Treg proliferation and survival, for instance, influence the function of liver kinase B 1, which, in turn, is an essential inducer of these pathways (415, 416). Further evidence for an involvement of mitochondrial metabolomic pathways derives from a study demonstrating an impaired Treg fitness due to an ablation of the mitochondrial complex III (417). Last, AMP activated protein kinase (AMPK)-dependent FAO is not only crucial for Treg generation, but also for their proliferation (**Figure 3****)** (418–420). While the metabolism of Tregs during homeostasis and proliferation is characterized by a balance of glycolysis and OXPHOS, a predominance of OXPHOS seems to be essential for the suppressive activity of Tregs. Thus, Tregs deficient for proteins that are crucial for mitochondrial metabolism, show significantly reduced suppressive functions and impaired allograft survival (421). Conversely, increased expression of genes that are important to OXPHOS leads to improved Treg suppressive function (421). Further support for a critical role of OXPHOS in the function of Tregs derives from the finding that liver kinase B 1, an enzyme involved in OXPHOS, is essential for the suppressive capacity of Tregs (417). Consequently, deficiency of liver kinase B 1 in mice was associated with a fatal, early-onset autoimmune disease (415). Moreover, also mTOR appears to be involved in the immunoregulatory activity of Tregs *via* induction of OXPHOS (422). Thus, for example, the suppressive activity of Tregs is associated with increased mTOR signaling. Furthermore, inhibition of mTOR reduced Treg-mediated suppression of T cell proliferation, while deficiency of mTOR in mice triggered autoimmune phenomena (422). In addition, the adequate immunosuppressive function of Tregs may also be associated with both enhanced FAO and fatty acid synthesis (423, 424). For example, stimulation of Tregs leads to increased FAO (418). On the other hand, inhibition of the mevalonate pathway, i.e. fatty acid synthesis, causes a restriction of the suppressive capacity of Tregs (425, 426). In contrast to OXPHOS, FAO, and fatty acid synthesis, enhancement of glycolysis could possibly even induce an impairment of the suppressive capacity of Tregs. For example, Treg-specific deficiency of phosphatase and tensin homolog (PTEN) in mice leads to spontaneous lupus-like disease accompanied by a reduction in the suppressive function of Tregs (427). Of note, increased glycolytic activity was detected in these Tregs, which in turn resulted in insufficient resolution of inflammatory activity EAE and subsequently to augmented disease progression (427). Further evidence for glycolysis-associated insufficiency of immunosuppressive activities derives from Tregs with an enhanced expression of glucose transporter 1, which showed an increased extent of glycolysis but impaired suppression (412). In human Tregs, however, maintenance of glycolytic activity was required for optimal suppressive properties of Tregs further highlighting the need for differential evaluation of immunometabolic processes in human and murine Tregs (411, 428).

In contrast, during migration of Tregs, there appears to be a shift towards glycolytic pathways. For example, hypoxia-inducible factor 1 α (HIF-1α)-deficient Tregs, which exhibit low levels of both glycolysis and enhanced OXPHOS, show increased suppressive properties but impaired migration (429). In mice, this subsequently led to reduced tumor growth and increased survival, likely due to enhanced anti-tumor immunity (429). Consistent with a prominent role of glycolytic processes during migration, stimuli such as CD28 triggering migration of Tregs induced an increased uptake of glucose and markedly enhanced glycolysis (430). Underlying mechanisms involve a PI3K-mTORC2-mediated pathway leading to an enhanced expression of glucokinase. In turn, Tregs deficient for this pathway showed diminished migration but preserved suppressive function. Consistently, Tregs from individuals with a mutation leading to enhanced glucokinase activity showed enhanced migration (430). In summary, a complex interplay and balance of different metabolic pathways underlie the different functional stages of Tregs activity.

Treg dysfunctionality is suggested to be the link between dysmetabolism or dysimmune reactions and pathogens. In fact, hyperglycemia suppressed HIF-1α effects on Tregs in aspergillus fumigatus infection (431). Together with defective NLRP3/IL-1*β* signal pathway, this led to a more widespread disbalance of Th1/Th2 ratio and Th17 cells (431). The HIF-1α-dependent glycolytic pathway stimulates Th17 differentiation and limits Treg development by promoting the function of RORγt (432, 433).

Targeting immunometabolism as an anti-inflammatory strategy has become an established method in clinical therapies (250, 434, 435). Recently, Palma et al. (436) reported on caloric restriction in mice promoting elimination of Mtb. By means of a multi-omic approach they defined changes in glycolysis next to reduction in fatty acid metabolism and mTOR activity as crucial effects of metabolic reprogramming in this condition (436). In addition to anti-inflammatory therapy, these findings on the metabolic properties of Tregs could also be used in tumor therapy to improve anti-tumor immunity *via* metabolic interventions. Thus, several aspects of the tumor microenvironment offer an advantage to Tregs, especially in comparison to Th1 or Th17 cells. For example, despite hypoxia, glucose shortness and acidosis, Tregs can compete with other T cells by increased expression of glucose transporters, enhanced glycolytic metabolism, and resistance to lactate-induced acidosis (391).

In the future, omic approaches might enable us to appreciate the complex interplay of immunorelevant pathological conditions and respective Treg functions. To this end, we hypothesize that the metabolic state is capable of reprograming immune responses, with Tregs in a key position, and Treg/Th17 ratio as a valuable judge for T cell function from blood-derived T cell analysis (437). Netea et al. (147) described the chance of so-called ‘innate immune training’: Metabolic shifts can cause epigenetic reprogramming in activated immune cells resulting in an innate immune memory resembling the adaptive immune system.

Strikingly, Arpaia et al. (438) showed that several metabolites produced by commensal bacteria, such as butyrate, short fatty acid and propionate (PA), represent a missing link between microbiota and the immune system promoting generation of pTregs. Interestingly, PA in particular led to a substantial increase in differentiation and proliferation of murine and human Tregs as well as a reduction in the differentiation of Th17 cells (367). Consistently, PA-treated mice showed an ameliorated EAE disease course accompanied by an increase in Treg frequency and IL-10 levels compared to controls. Of note, even application of Tregs derived from mice pre-treated with PA attenuated the clinical symptoms of EAE (367). In addition to these findings obtained from EAE, clinical data from MS patients also pointed in this direction. Not only decreased PA serum and feces levels were found in all disease entities of MS, especially after the first relapse (439). Rather, the add-on administration of PA in MS patients led to a reversal of the Treg/Th17 imbalance, i.e. to an induction of Tregs and a reduction of pro-inflammatory Th1 and Th17 cells. Furthermore, these patients even showed a lower annual relapse rate, a reduced disability progression, and a minor brain atrophy. In line, the microbiome of these patients also showed an upregulation of Treg-inducing genes following PA treatment. These findings indicate a large potential benefit of PA therapy (439).

## 3 Discussion

In conclusion, microorganisms and immune responses to pathogens are closely interrelated: Programming of the immune system determines the effectiveness of pathogen elimination or their persistence, respectively. *Vice versa*, pathogens can affect immune responses. Specifically, the balance between T cell subpopulations, in particular the Treg/Th17 ratio, influences the defense against pathogens. In this context, Tregs exhibit a Janus-faced nature: Pronounced Treg activation correlates with a restricted and tissue-protective immune response but can result in pathogen persistence. In contrast, impaired Treg activity, together with Th1 and Th17 responses, can effectively eliminate pathogens but can also result in substantial collateral tissue damage. Consistent with this, mechanisms potentially reprogramming and modulating Treg responses, and their pathogenic conversion are currently intensely investigated. The pathogen itself can impact frequency and function of Tregs by a variety of mechanisms. It can alter immune responses to escape immune activation or even infiltrate immune cells using them as Trojan horses to infect the environment. Metabolic conditions like hyperglycemia or diabetes correlating with high fat diet in animal experiments can reprogram those immune responses by a variety of complex mechanisms not yet fully understood and in urgent need for further investigation. In this context, certain commensal microbiota alter the immune balance, especially the Treg/Th17 ratio.

There is substantial evidence that the interplay of pathogenic infections and immune processes are crucial in the pathogenesis of autoimmune disorders. Several pathogens were shown to be critically involved in MS immunopathophysiology by modulating the immune response. The best evidence is available for host-detrimental effects of EBV and HERVs which seem to fuel neuroinflammation. These effects are induced by different mechanisms such as bystander activation, molecular mimicry, superantigen-induced promotion of the T cell response, and epitope spreading. However, apart from these deleterious effects, protective effects have been demonstrated for numerous parasites, especially helminths, and also H. pylori. Such insights into the interplay of infection and MS have subsequently led to numerous translational approaches aimed at inhibiting deleterious and promoting favorable interactions. In addition to cell-based immunotherapies targeting EBV-infected B cells, the application of the monoclonal antibody GNbAC1 inhibiting HERV-W ENV seems particularly promising. Interestingly, GNbAC1 not only leads to a suppression of pro-inflammatory signaling pathways but also to possible critically desired neuroregenerative effects. The underlying mechanisms probably include improved OPC differentiation and a neutralization of pro-inflammatory myeloid cells. Accordingly, results of the ongoing phase IIb ProTect-MS study are awaited with great interest.

Further therapeutic opportunities might be provided by a closer understanding of the interaction of the intestinal microbiome with Tregs thereby influencing the Treg/Th17 balance. Dysregulation of this balance or of the microbiome in the sense of dysbiosis promotes the occurrence of autoimmune diseases. Furthermore, the composition of the microbiome seems to have enormous effects on the phenotype of Tregs. However, these effects are not only local, but lead to a systemic modulation of the immune response with both potentially unfavorable and favorable consequences. Nevertheless, deeper insights into this interplay are necessary to develop therapeutic strategies to take advantage of this knowledge. Possible candidates for this are Clostridium species or Bacteroides fragilis, which provide favorable immune modulatory properties with regard to autoimmunity by promoting regulatory capacities of Tregs.

Impacting both innate and adaptive immune responses, future research should identify the most relevant pathogen species as well as define molecular mechanisms and substrates underlying reprogramming of the immune balance. Such new insights could be used to therapeutically induce or modify Tregs to treat autoimmune diseases and immune responses in general. Hence, multi-omic approaches may unravel the interplay between microorganism-modulated Treg function and autoimmune neuroinflammation. Understanding the underlying molecular mechanisms could further contribute to the development of targeted therapies.

## Author Contributions

CS and TR conceptualized the outline of the manuscript. CS and NH wrote the original draft of the manuscript. CS and NH were responsible for graphical illustrations. SB, CN, DK, KP, SM, and TR reviewed and edited the manuscript. All authors contributed to the article and approved the submitted version.

## Conflict of Interest

The authors declare that the research was conducted in the absence of any commercial or financial relationships that could be construed as a potential conflict of interest.

## Publisher’s Note

All claims expressed in this article are solely those of the authors and do not necessarily represent those of their affiliated organizations, or those of the publisher, the editors and the reviewers. Any product that may be evaluated in this article, or claim that may be made by its manufacturer, is not guaranteed or endorsed by the publisher.

## Glossary

**Table:**

AMPK	AMP activated protein kinase
APCs	antigen-presenting cells
C. pneumoniae	Chlamydia pneumoniae
CNS	central nervous system
CSF	cerebrospinal fluid
CTLA-4	cytotoxic T-lymphocyte-associated protein 4
DCs	dendritic cells
EAE	experimental autoimmune encephalomyelitis
EBNA-1	EBV nuclear antigen 1
EBV	Epstein-barr virus
FAO	fatty axid oxidation
Foxp3	forkhead box protein P3
FV	friend retrovirus
GEL	gadolinium-enhancing lesion
GITR	glucocorticoid-induced tumor necrosis factor receptor
H. pylori	Helicobacter pylori
HBV	hepatitis B virus
HCV	hepatitis C virus
HERV	human endogenous retrovirus
HHV-6	human herpes virus 6
HIF-1α	hypoxia-inducible factor 1 α
HIV	human immunodeficiency virus
HSV	herpes simplex virus
IDO	indoleamine 2,3-dioxygenase
IFNγ	interferon γ
IL	interleukin
MBP	myelin basic protein
MRI	magnetic resonance imaging
MS	multiple sclerosis
Mtb	mycobacterium tuberculosis
mTOR	mechanistic target of rapamycin
NK cells	natural killer cells
OPC	oligodendrocyte precursor cell
OXPHOS	oxidative phosphorylation
PA	propionate
PD-L1	programmed death-1 ligand 1
PKA	protein kinase A
PTEN	phosphatase and tensin homolog
pTregs	peripherally induced regulatory T cells
RORγt	retinoic acid-related orphan receptor gamma t
RRMS	relapsing-remitting MS
SCFAs	short-chain fatty acids
STAT	signal transducer and activator of transcription
Tconv	conventional T cells
TCR	T cell receptor
TGFb	tumor growth factor b
Th	T helper
TIGIT	T cell immunoreceptor with Ig and and immunoreceptor tyrosine-based inhibitory motif domains
TLR	Toll-like receptor
TNF	tumor necrosis factor
Tregs	regulatory T cells
tTregs	thymus-dervived regulatory T cells
